# Alzheimer's disease recognition via long-range state space model using multi-modal brain images

**DOI:** 10.3389/fnins.2025.1576931

**Published:** 2025-05-19

**Authors:** Ziyin Ren, Meng Zhou, Sadia Shakil, Raymond Kai-Yu Tong

**Affiliations:** Department of Biomedical Engineering, The Chinese University of Hong Kong, Hong Kong, Hong Kong SAR, China

**Keywords:** Alzheimer's disease, long-range sequential modeling, mild cognitive impairment, multi-modal brain images, multi-modality integration

## Abstract

As a persistent neurodegenerative abnormality, Alzheimer's disease (AD) is affecting an increasing number of elderly people. The early identification of AD is critical for halting the disease progression at an early stage. However, the extraction and fusion of multi-modal features at different scales from brain images remains a challenge for effective AD recognition. In this work, a novel feature fusion long-range state space model (FF-LSSM) model is suggested for effective extraction and fusion of multi-level characteristics from scannings of MRI and PET. The FF-LSSM can extract whole-volume features at every scale and effectively decide their global dependencies via adopted 3D Mamba encoders. Moreover, a feature fusion block is employed to consolidate features of different levels extracted by each encoder to generate fused feature maps. A classifier is cascaded at the end, using the fused features to produce the predicted labels. The FF-LSSM model is optimized and evaluated using brain images of subjects from the ADNI dataset. The inference result on the testing set reveals the FF-LSSM accomplishes a classification ACC of 93.59% in CN vs. AD and 79.31% in sMCI vs. pMCI task, proving its effectiveness in disease classification. Finally, the introduction of the Grad-CAM method illustrates that the implied FF-LSSM can detect AD- and MCI-related brain regions effectively.

## 1 Introduction

Alzheimer's disease (AD) is an irreversibly progressive central nervous system degenerative disorder, which has become the major cause of most dementia among old citizens: there are over fifty million AD sufferers worldwide, according to statistics (Dadar et al., 2017; Zou et al., 2024). As patients progress to AD, their cognitive abilities and memory gradually decline. Mild cognitive impairment (MCI) is the initial stage of this decline. Depending on its severity and progression, MCI can be further classified as either the earlier stable MCI (sMCI) stage or the later progressive MCI (pMCI) stage. After the MCI stage, as the continuous neurodegeneration, patients will eventually progress to AD, leading to a complete loss of self-care capabilities (Scheltens et al., 2021; Colom-Cadena et al., 2020). The governments and families of AD patients will face significant financial burdens due to the disease's high incidence. Nowadays, there is no effective treatment for AD; current medications only work to lessen symptoms and halt the disease's progression (Pawar et al., 2025). However, early diagnosis of Alzheimer's remains essential as it can provide early intervention and thus prohibit AD progression at an early stage. Recently, the development of artificial intelligence (AI) has made it possible to detect AD using deep learning (DL) methods.

Brain imaging can provide rich information about patients' pathological and anatomical status. Currently, many kinds of brain imaging methods have been used in AD detection. The ability of magnetic resonance imaging (MRI) to detect subtle structural alterations in the brain, including AD biomarkers like atrophy in the hippocampus, frontal lobe, and temporal lobe, makes it a common diagnostic tool for AD (Hunter et al., 2024). Due to this capability, several tools have been proposed using structural MRI for AD detection in past years (Lin et al., 2018; Mofrad et al., 2021; Inan et al., 2024). However, structural AD biomarkers often appear after the disease has progressed; when AD progression is just getting started, these biomarkers are usually undetectable. Another common neuroimaging method for identifying AD is Positron Emission Tomography (PET). During PET imaging, a radiotracer is administered into the patient's body, and metabolic activity can be measured by observing the accumulation of radio substances. In practice, different radiotracers will be chosen according to distinct imaging purposes. Among them, Fluorine-18 fluorodeoxyglucose (18F-FDG) is one of the most popular radiopharmaceuticals. As a glucose analog, the uptake of 18F-FDG is a marker for glucose consumption and thus can highlight active brain regions during PET imaging (Li and Tang, 2015). Recent research has shown that brain 18F-FDG PET can detect signs of AD neuropathy in people with MCI earlier than brain MRI, making it an informative tool for early AD recognition (Nobili et al., 2018). Therefore, there are an increasing number of methods based on 18F-FDG PET modality for AD screening have been proposed (Chen et al., 2022; Duan et al., 2023; Rogeau et al., 2024). However, PET imaging has limitations in terms of resolution and signal-to-noise ratio, which makes it difficult to obtain information on small scales. A potential improvement is to fuse features from two imaging modalities. It is possible to combine PET's superior early-stage AD detection capabilities with the high spatial resolution of MRI.

The fusion of multi-modal features for computer-aided diagnosis has become increasingly popular, many approaches have been suggested in recent years for detecting Schizophrenia (Kanyal et al., 2024), ADHD (Sethu and Vyas, 2020; Yao et al., 2021), ASD (Wang Q. et al., 2023; Abbas et al., 2023), and also AD (Zuo et al., 2023a,b, 2024a,b; Zong et al., 2024). The current multi-modal approaches for AD identification can be broadly divided into two types: those that rely on conventional machine learning (ML) and those that depend on DL. ML-based methods usually use the constructed classifier for disease recognition after extracting features from the brain's pre-defined regions of interest (ROIs). For example, a linear support vector machine is adopted by Zhang et al. (2011) to incorporate multi-modal features such as cerebrospinal fluid (CSF) biomarkers and 93 ROIs' MRI tissue volumes and PET intensity values for AD classification. The work in Tong et al. (2017) utilized a framework based on non-linear graph fusion to integrate biomarkers from different modalities and adopted a random forest algorithm to distinguish MCI, AD, and cognitive normal (CN) control. A multi-modal progressive graph-based transductive learning method is suggested by Wang et al. (2017) that can gradually learn latent intrinsic representations from MRI and PET imaging to achieve optimized dementia classification. The work in Shi et al. (2019) suggested diagnosing AD and MCI using the interaction of coupled representations from MRI and PET features. DL methods become more and more prevalent in recent years because, in contrast to more conventional ML approaches, DL-based approaches can not only automatically learn representations from the input without manually designed features, but they can also extract characteristics from the latent space to capture the abstract expression of data. The work in Lu et al. (2018), for example, suggested a multi-scale deep neural network (DNN) that uses MRI and PET modalities to predict the conversion to MCI and AD. By identifying the interaction between the multi-modal images, Zhang and Shi (2020) proposed a feature fusion model based on the residual network and attention mechanism. The work by Fang et al. (2020) designed an ensemble classifier for AD prediction by fusing each modality's slice-wise probabilistic score generated by three different deep convolution neural network (CNN) models. The work by Hu et al. (2023) utilized VGG-16-based CNN and multi-head self-attention mechanism to build a VGG-TSwinformer model using T1- and T2-weighted MRI for MCI transition prediction. However, many models concentrate solely on modality-shared representations or modality-specific information while neglecting feature integration. Additionally, most previous methods divide input images into patches without considering the extraction and amalgamation of features at various scales from the voxel to the global level.

In the analysis of high-dimensional medical images, as the input size increases, the computational complexity and convergence time of traditional architectures, such as Transformer, grow drastically, which limits their application potentials. Recently, a novel DL framework, Mamba, has been proposed to tackle the challenge of modeling long sequences (Gu and Dao, 2023). The basic structure of Mamba is based on the state space model (SSM) (Kalman, 1960), by introducing a selection mechanism and a hardware optimization method, Mamba can capture global long-range dependencies and achieve higher training and inference efficiency. Although it was originally designed to handle long sequence modeling problems, the linear complexity of SSM can effectively handle the computational challenges brought by high-dimensional medical images. Many studies have incorporated Mamba frameworks into computer vision and medical image applications, such as the Vision Mamba (Zhu et al., 2024), which introduces the Vim block for enhanced location-aware visual understanding. The work by Ma et al. (2024) proposed the U-Mamba that integrates the Mamba layer into the nnUNet encoder for 2D medical image segmentation. SegMamba is an innovative architecture incorporating a 3D Mamba encoder for segmenting 3D medical images (Xing et al., 2024). The 3D Mamba encoder can convert an input 3D feature map to three long sequences in different orders and thus enable the SSM to model 3D features. Like its extraordinary performance in long-sequence modeling of large language models, it is observed that the SSM can not only extract whole-volume 3D features at every scale but also effectively decide their global dependencies. All these works together demonstrate the great potential of the Mamba architecture in DL based medical imaging analysis. Using high-resolution brain imaging data, such as MRI and PET, to diagnose AD often requires capturing the most subtle status changes over long sequences and processing the relationship between multiple potential degeneration locations, which is a task suitable for introducing SSM-based models.

To tackle the challenge of global dependencies extraction and multi-scale feature fusion, in this research, we propose a Feature Fusion Long-range State Space Model (FF-LSSM) for AD detection and MCI conversion prognosis using MRI and PET images. According to our awareness, it is the first DL model that incorporates the SSM into AD classification. The FF-LSSM takes MRI and PET images of each subject as input. Features from two modalities are extracted separately by two Mamba encoders based on the Tri-orientated Spatial Mamba (TSMamba) block (Xing et al., 2024). A feature fusion block based on the dynamic feature fusion (DFF) module (Yang et al., 2024) is introduced to integrate multi-scale features from distinct modalities. Then, a classifier is introduced to generate the predicted labels from fused feature maps. For better model explainability, we also employ the gradient-weighted class activation mapping (Grad-CAM) method to plot the brain areas that contribute the most to dementia classification.

The remaining paragraphs of this paper are expanded in the sequence outlined below. In Section 2, we describe subjects and image pre-processing methods applied in our research. In Section 3, we thoroughly illustrate the suggested DL model. In Section 4, we clarify the model implementation detail and experimental outcomes. In Section 5, we collate the obtained results with those reported in previous papers and discuss the proposed method's advantages and restrictions. In Section 6, a summary conclusion is given by us.

## 2 Materials

### 2.1 Dataset

All data applied in this research is acquired from the public Alzheimer's Disease Neuroimaging Initiative (ADNI) dataset, which is a multi-center research program with the goal of discovering and validating biomarkers for AD (Jack et al., 2008). In ADNI, thousands of people from throughout North America were brought together for clinical assessments and brain imaging. This study uses MP-RAGE T1-weighted MRI and 18F-FDG PET images from ADNI studies for model training and evaluation. Data is obtained from the baseline MRI and PET acquisition of 656 individuals, which encompasses 197 from CN group, 162 from sMCI group, 104 from pMCI group, and 193 from AD group. We obtained labels for sMCI and pMCI groups from published data of one previous study (Gao et al., 2021), which are defined based on whether a subject progressed to AD within 36 months after the initial assessment. Since not all volunteers in the ADNI database have data from all modalities, we only screened out those who had both MRI and PET brain images as subjects for this study. The detailed specifics of the selected subjects, including their gender, age, clinical dementia rating (CDR), and mini-mental state examination (MMSE), are itemized in Table 1.

**Table 1:** Demographic information and clinical scores of studied groups (mean ± std).

**Group**	**Gender (F/M)**	**Age (Years)**	**CDR**	**MMSE**
CN	102/95	74.1 ± 5.6	0.0 ± 0.1	29.1 ± 1.1
sMCI	55/107	74.6 ± 7.3	1.4 ± 0.8	27.5 ± 1.8
pMCI	43/61	74.7 ± 6.6	1.9 ± 1.0	26.8 ± 1.7
AD	96/97	74.9 ± 8.0	4.5 ± 1.7	23.2 ± 2.2

### 2.2 Image pre-processing

**Figure 1:**
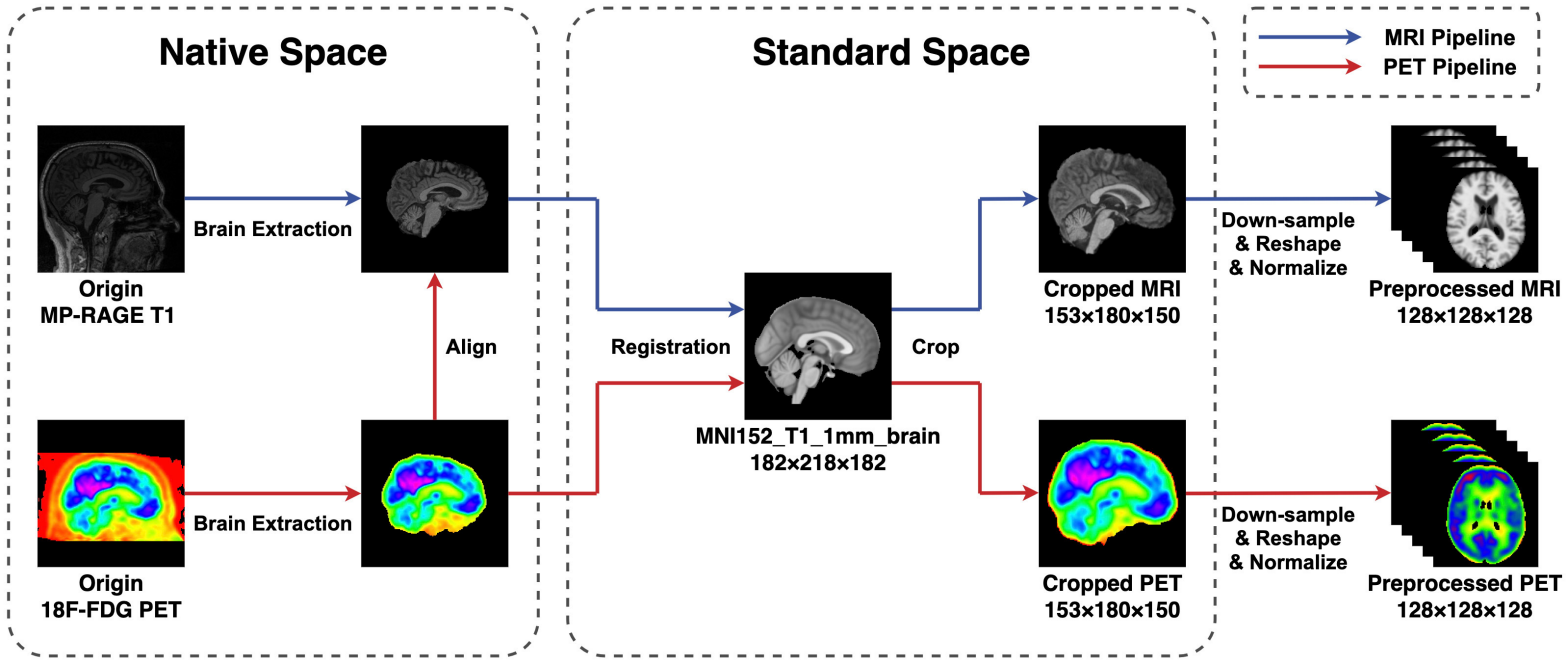
The pipeline of image preprocessing applied in this research. The BET method is applied for extracting the brain, and the FLIRT algorithm is used for linear registration. To reduce the computation complexity, all images are first cropped and then down-sampled and reshaped to a size of 128 × 128 × 128 mm. At last, all voxel values are normalized between 0 and 1.

The 18F-FDG PET images provided by ADNI are all in a slice-wise DICOM format, while the MP-RAGE MRI are in 3D NIFTI format. To ensure the uniformity of the file type and ease subsequent steps, we first use the SPM12 toolbox (Penny et al., 2011) to alter PET files from DICOM to NIFTI format. Subsequently, both MRI and PET images are read and manipulated by script commands of FSL 6.0.7 (Smith et al., 2004; Woolrich et al., 2009; Jenkinson et al., 2012). The pre-processing steps follow a standard pipeline. First, the brain extraction is performed using the BET algorithm (Smith, 2002) to strip the skull from the native space of each subject. Then, all MRI images are linearly registered to the MNI152 standard space by utilizing the FLIRT algorithm (Jenkinson et al., 2002). Afterward, the PET images are first aligned to their corresponding MP-RAGE files in the individual space and then transferred into the template space using the transformation matrix generated in the previous step. After all scannings are registered to the standard space, we remove zero-valued voxels close to the edges and leave the center of the volume with a size of 153 × 180 × 150 mm. The remaining volumes are then down-sampled and resized to a cubic shape of 128 × 128 × 128 mm to reduce the computational complexity. At last, all voxel values in each volume *I* are normalized between 0 and 1 to facilitate the model training and evaluation. The image pre-processing pipelines are summarized in Figure 1.

## 3 Method

**Figure 2:**
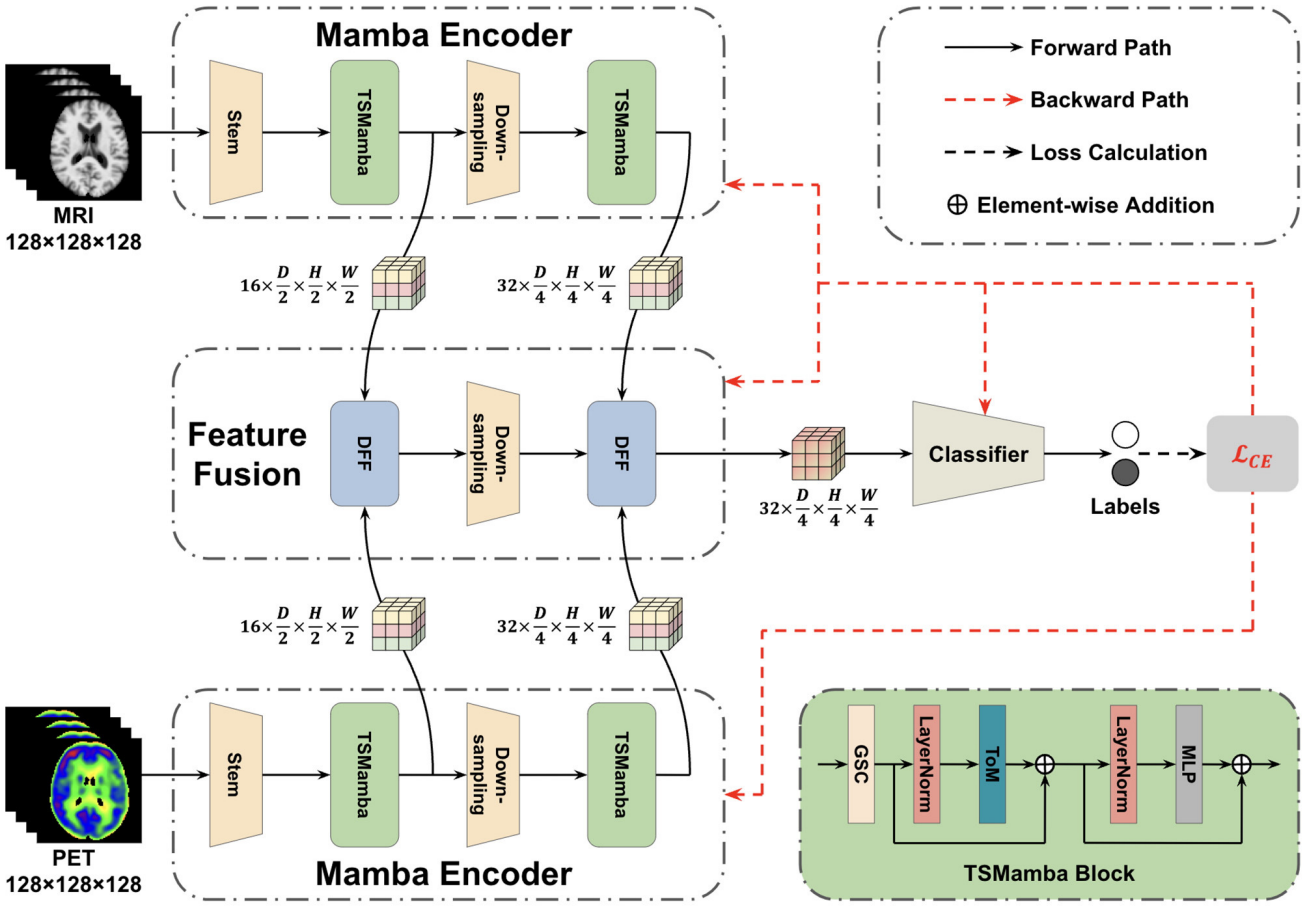
Architecture of the suggested FF-LSSM framework. The FF-LSSM contains two 3D Mamba encoders for feature extraction, one feature fusion block to fuse multi-modal features, and one classifier to generate predicted labels.

After the data pre-processing steps are finished, an FF-LSSM framework with four parts is proposed for multi-modal AD classification, as demonstrated in Figure 2. Our suggested DL framework is comprised of the subsequent four elements: (1) two 3D Mamba encoders constructed with down-sampling layers and TSMamba blocks to obtain and combine multi-level features from the voxel to the global level, (2) one feature fusion block based on the DFF module, and (3) one classifier to generate desired labels. The following subsections describe each block's specifics in detail.

### 3.1 Mamba encoder

As the backbone of the proposed model, two Mamba encoders (Xing et al., 2024) are adopted separately to obtain latent features from the pre-processed MRI and PET volumes. In the Mamba encoder, the input 3D volume first passes a stem 3D convolution layer that has a large kernel size of 7, a stride length of 2, and a padding of 3. In this research, we empirically set the output channel number to 16 for this stem layer. Thus, the stem layer can extract an initially down-sampled feature map $z_{0}\in 16\times \frac{D}{2}\times \frac{H}{2}\times \frac{W}{2}$ for each input volume *I*∈1 × *D*×*H*×*W*.

Following the stem layer, the scaled feature map *z*_0_ is fed into a TSMamba block. As demonstrated in Figure 2, the TSMamba block comprises a Gated Spatial Convolution (GSC) module followed by a Tri-orientated Mamba (ToM) module and a multi-layer perceptron (MLP). To facilitate the training and convergence of the model, residual connections and the layer normalization (LN) operation are also introduced. The GSC module in the TSMamba block is applied to preliminarily process spatial features and relationships in input feature maps. The output of GSC is divided into two parts, one is retained as the residual link, and the other one is fed into a ToM module after the LN operation. Unlike the original Mamba module that is proposed for modeling long time-series 1D sequences, the ToM module is a modified Mamba layer that can calculate feature dependencies from three directions, making it applicable for modeling 3D volumes. For a given 3D input feature map *x*, the ToM module first flattens it into voxel sequences in three directions, representing the forward *x*_*f*_, reverse *x*_*r*_, and inter-slice *x*_*s*_ sequences. Each sequence is processed by an independent Mamba module to extract features and relationships at each level. Finally, the outputs of three Mamba modules are added to achieve the feature integration, which computational descriptions can be denoted as (Xing et al., 2024):


(1) $$\begin{array}{l} ToM\left(x\right)=M\left(x_{f}\right)+M\left(x_{r}\right)+M\left(x_{s}\right) \end{array}$$

where *M* means the 1D Mamba module. Output of the ToM module is also split into two sections, one is fed into an MLP after the LN operation, and the other one is used as the residual connection. For a given input feature map *y*_*i*_, the computational expression of the TSMamba block is as follows (Xing et al., 2024):


(2) $$\begin{array}{l} \hat{y_{i}}=GSC\left(y_{i}\right) \end{array}$$

(3) $$\begin{array}{l} \tilde{y_{i}}=ToM\left(LN\left(\hat{y_{i}}\right)\right)+\hat{y_{i}} \end{array}$$

(4) $$\begin{array}{l} y_{i+1}=MLP\left(LN\left(\tilde{y_{i}}\right)\right)+\tilde{y_{i}} \end{array}$$

where *y*_*i*+1_ represents the output. In this work, we set the input and output channels of all layers in a TSMamba block to be the same, so for an input $z_{0}\in 16\times \frac{D}{2}\times \frac{H}{2}\times \frac{W}{2}$, an output $z_{1}\in 16\times \frac{D}{2}\times \frac{H}{2}\times \frac{W}{2}$ of the same size is returned. Details of the TSMamba block can be found in the salient work of Xing et al. (2024) and will not be repeated here.

For further down-sampling and feature extraction, the feature extracted by the first TSMamba block then passes through a down-sampling 3D convolution layer of kernel 3, stride 2, and padding 1. For the extracted feature of the first TSMamba module $z_{1}\in 16\times \frac{D}{2}\times \frac{H}{2}\times \frac{W}{2}$, the scaled feature $z_{2}\in 32\times \frac{D}{4}\times \frac{H}{4}\times \frac{W}{4}$ is generated by this down-sampling layer. Then, the scaled feature passes through the second TSMamba module for further feature interaction and feature fusion. The output of the second TSMamba module can be denoted as $z_{3}\in 32\times \frac{D}{4}\times \frac{H}{4}\times \frac{W}{4}$.

### 3.2 Feature fusion block

**Figure 3:**
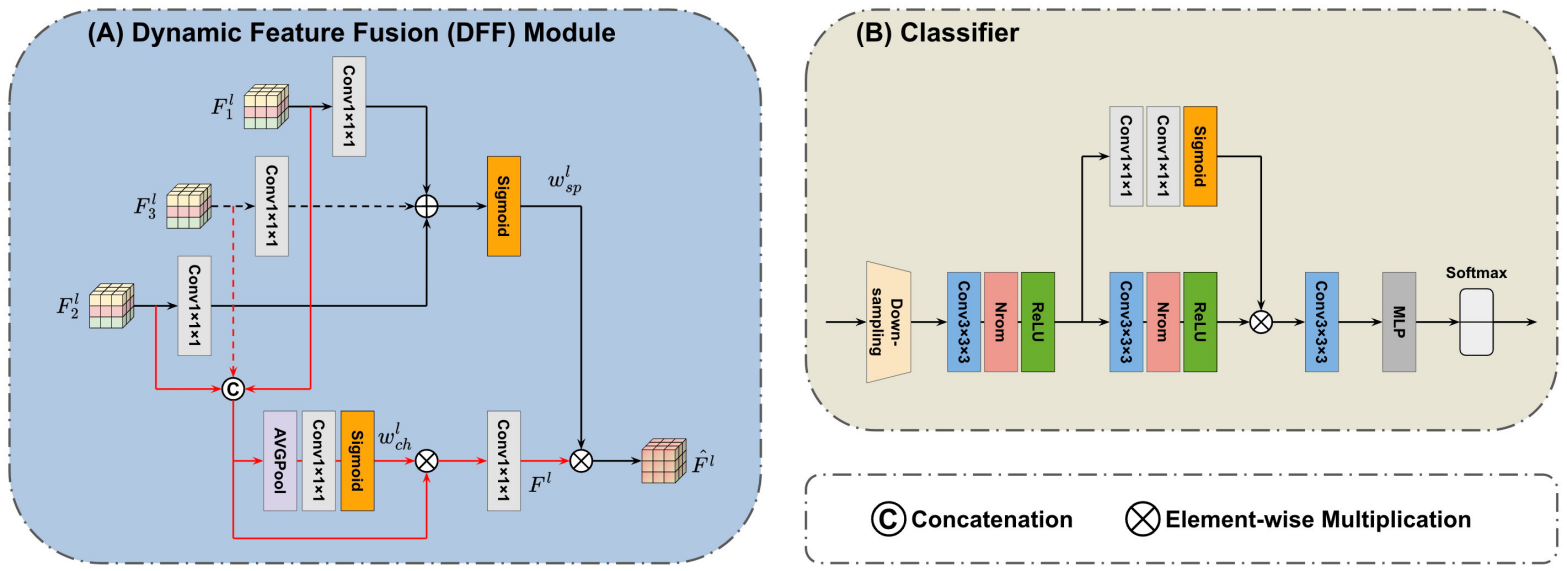
Architecture of the applied **(A)** DFF module (Yang et al., 2024) and **(B)** classifier. The DFF consists of two paths. In one path, the global channel information $w_{ch}^{l}$ is extracted from concatenated input features. In another path, the global spatial information $w_{sp}^{l}$ is extracted from input features by separate convolution layers. The final output ${\hat{F}}^{l}$ integrates both the channel and spatial information. The superscript *l* indicates the *l*-th DFF module. The classifier first adopts a down-sampling layer to reshape the feature map. The scaled features are then processed by three convolution layers, one self-attention branch, and an MLP to generate the predicted label.

The DFF module (Yang et al., 2024) is introduced in the feature fusion block to fuse features extracted by the Mamba encoders. The principle of DFF is using global information from the input itself as a guide to adaptively fuse local features at multi-scales. The progress of feature fusion involves the dynamic selection of essential features by considering their global information. The outputs of the first TSMamba block in both MRI and PET Mamba encoders are used as the input of the first DFF module. We can denote two input features of the first DFF module as $F_{1}^{1}\in 16\times \frac{D}{2}\times \frac{H}{2}\times \frac{W}{2}$ and $F_{2}^{1}\in 16\times \frac{D}{2}\times \frac{H}{2}\times \frac{W}{2}$. As shown in Figure 3A, a concatenation operation is performed on the channel dimension of inputs $F_{1}^{1}$ and $F_{2}^{1}$, resulting in the feature with a dimension of $32\times \frac{D}{2}\times \frac{H}{2}\times \frac{W}{2}$. By letting the concatenated input pass through an average pooling operation, a convolution layer with 32 output channels, and a Sigmoid activation in sequence, the global channel information $w_{ch}^{1}$ that describes the importance of features can be extracted (Yang et al., 2024):


(5) $$\begin{array}{l} w_{ch}^{1}=Sigmoid\left(c^{1\times 1\times 1}\left(AVGPool\left[F_{1}^{1};F_{2}^{1}\right]\right)\right) \end{array}$$

where *c* stands for the convolution layer. The product of $w_{ch}^{1}$ and the concatenated input then passes through a 1 × 1 × 1 convolution layer with 16 output channels to make the channel number the same as the original one. In this way, the global channel information guides the retaining of highlighted features while removing useless interference. The following equation can represent the process (Yang et al., 2024):


(6) $$\begin{array}{l} F^{1}=c^{1\times 1\times 1}\left(w_{ch}^{1}\cdot \left[F_{1}^{1};F_{2}^{1}\right]\right) \end{array}$$

Besides channel information, spatial information is also crucial in the DFF process. While generating global channel information, $F_{1}^{1}$ and $F_{2}^{1}$ also pass through two 1 × 1 × 1 convolution layers with 16 output channels, respectively. The summation of these two convolution layers' outputs then passes through a Sigmoid activation to generate the global spatial information *w*_*sp*_ (Yang et al., 2024):


(7) $$\begin{array}{l} w_{sp}^{1}=Sigmoid\left(c^{1\times 1\times 1}\left(F_{1}^{1}\right)+c^{1\times 1\times 1}\left(F_{2}^{1}\right)\right) \end{array}$$

The global spatial information is then multiplied with *F*^1^, which integrates the spatial and channel information, thereby highlighting critical locations on the feature map. Thus, the first DFF module's output can be calculated by Yang et al. (2024):


(8) $$\begin{array}{l} {\hat{F}}^{1}=w_{sp}^{1}\cdot F^{1} \end{array}$$

The output of the first DFF module then passes through the same down-sampling 3D convolution layer. This convolution layer resizes the feature map to make it have a dimension of $32\times \frac{D}{4}\times \frac{H}{4}\times \frac{W}{4}$, the same size as the outputs of the second TSMamba module in both Mamba encoders. The outputs of each Mamba encoder's second TSMamba module are then supplied into the second DFF module together with the fused feature map generated by the first DFF module. For the second DFF module, which has three inputs, Equations 5–8 can be replaced by:


(9) $$\begin{array}{l} w_{ch}^{2}=Sigmoid\left(c^{1\times 1\times 1}\left(AVGPool\left[F_{1}^{2};F_{2}^{2};F_{3}^{2}\right]\right)\right) \end{array}$$

(10) $$\begin{array}{l} F^{2}=c^{1\times 1\times 1}\left(w_{ch}^{2}\cdot \left[F_{1}^{2};F_{2}^{2};F_{3}^{2}\right]\right) \end{array}$$

(11) $$\begin{array}{l} w_{sp}^{2}=Sigmoid\left(c^{1\times 1\times 1}\left(F_{1}^{2}\right)+c^{1\times 1\times 1}\left(F_{2}^{2}\right)+c^{1\times 1\times 1}\left(F_{3}^{2}\right)\right) \end{array}$$

(12) $$\begin{array}{l} {\hat{F}}^{2}=w_{sp}^{2}\cdot F^{2} \end{array}$$

Since the second DFF module has an additional input path compared with the first one, the channel number after concatenation becomes 96. Therefore, in the branch that calculates the global channel information, the convolution layers' input and output channels are adjusted appropriately to make sure the size of the output stays constant. The final output for the feature fusion block can be denoted as $z_{4}\in 32\times \frac{D}{4}\times \frac{H}{4}\times \frac{W}{4}$.

### 3.3 Classifier

After the feature fusion block, a classifier is accessed to generate the predicted result from combined and learned feature maps. As demonstrated by Figure 3B, the input feature map of the classifier first goes through another down-sampling 3D convolution layer, which produces the further rescaled feature map denoted as $z_{5}\in 64\times \frac{D}{8}\times \frac{H}{8}\times \frac{W}{8}$. Subsequently, this feature map asses through two convolution blocks (each consisting of one 3 × 3 × 3 convolution, one normalize, and one ReLU layer) and then one last 3 × 3 × 3 convolution layer followed by an MLP. The fully connected MLP outputs two classes with a Softmax function to produce output labels. Moreover, the classifier adopts a self-attention branch with two cascaded 1 × 1 × 1 convolution layers followed by the Sigmoid activation. This attention branch can highlight important features and thus facilitate modeling multi-level and long-range correlations.

## 4 Experiments and results

### 4.1 Implementation

We deploy the suggested DL model leveraging the Pytorch toolkit in a Python 3.10.14 environment. The experimental environment is built on an Ubuntu 18.04 platform with an NVIDIA GeForce RTX3090 GPU accelerated by the CUDA framework. All models are trained and evaluated on two tasks of binary classification: AD detection (CN vs. AD) and MCI prognosis (sMCI vs. pMCI). In both tasks, subjects from the corresponding groups are randomly assigned to the training and testing sets in a 4:1 ratio. The same training and testing sets are used for all models' optimization and inference processes. In the model training, we adopt an Adam optimizer, its learning rate is set to 1 × 10^−4^, and a weight decay parameter of 0.02 is added. The training batch size is set to 4, i.e., the brain images of 4 subjects. We define the cross-entropy between the model's output and the subject's real label as the loss function:


(13) $$\begin{array}{l} L_{CE}\left(p,q\right)=-\overset{n}{\underset{i=1}{\sum }}p_{i}\log q_{i} \end{array}$$

where *n* implies the class number, *p*_*i*_ refers to the real label, and *q*_*i*_ indicates the label predicted by the model. To quantitatively measure model performance, we calculate four commonly used indicators on the test set, which are accuracy (ACC), sensitivity (SEN), specificity (SPE), and area under the curve (AUC).

### 4.2 Effectiveness of disease classification

To investigate the efficacy of the suggested FF-LSSM in AD detection, we first examine its performance on CN vs. AD task. Meanwhile, to verify the advantages of multi-modality methods over uni-modality ones, we also evaluate variant models using only MRI or PET as input. In the variant uni-modality models, we remove one Mamba encoder from them. Since one input path is eliminated, the channel number of their DFF module's convolution layers is adjusted accordingly. Besides, we also implement a SOTA method, the pathwise transfer dense convolution network (PT-DCN) (Gao et al., 2021), on the same training and testing dataset for comparison. Since the input size of the original PT-DCN is different from ours, we change part of its structures and try our best effort to restore its deployment environment for a fair comparison. On the left side of Table 2, we list the results of the aforementioned experiments. Experimental results show that the MRI-based uni-modality model performs the worst, the PET variant performs slightly better, and the proposed FF-LSSM has the best classification performance, achieving 93.59% in ACC. Credit to better abilities of modeling high-dimension medical images and feature integration, the propsed FF-LSSM also outperforms the previous PT-DCN that based on traditional CNN.

**Table 2:** Comparison of results for classification of CN vs. AD and sMCI vs. pMCI tasks using uni- and multi-modal images as input.

**Modality**	**CN vs. AD (%)**				**sMCI vs. pMCI (%)**			
	**ACC**	**SEN**	**SPE**	**AUC**	**ACC**	**SEN**	**SPE**	**AUC**
MRI	88.46	86.84	90.00	92.96	72.41	69.56	74.29	69.07
PET	91.03	89.47	92.50	95.10	77.59	73.91	**80.00**	74.91
PT-DCN	92.31	89.74	94.87	95.20	77.59	78.26	77.14	79.13
FF-LSSM	**93.59**	**92.11**	**95.00**	**96.18**	**79.31**	**86.96**	74.29	**80.62**

Bold values indicate the highest value of each metric.

Subsequently, we conducted the same experiment on the sMCI vs. pMCI task to evaluate the effectiveness of models in MCI prognosis, and the corresponding results are enumerated on the right side of Table 2. In addition to the PET-based variant showing better SPE, the experimental results on the sMCI vs. pMCI task are consistent with the previous CN vs. AD one: FF-LSSM shows the highest 79.31% ACC, followed by the PET-based model and PT-DCN, and then the MRI-based model. This demonstrates the effectiveness of FF-LSSM in the prognosis of MCI and further proves the significance of the multi-modality integration technique.

### 4.3 Ablation study

**Figure 4:**
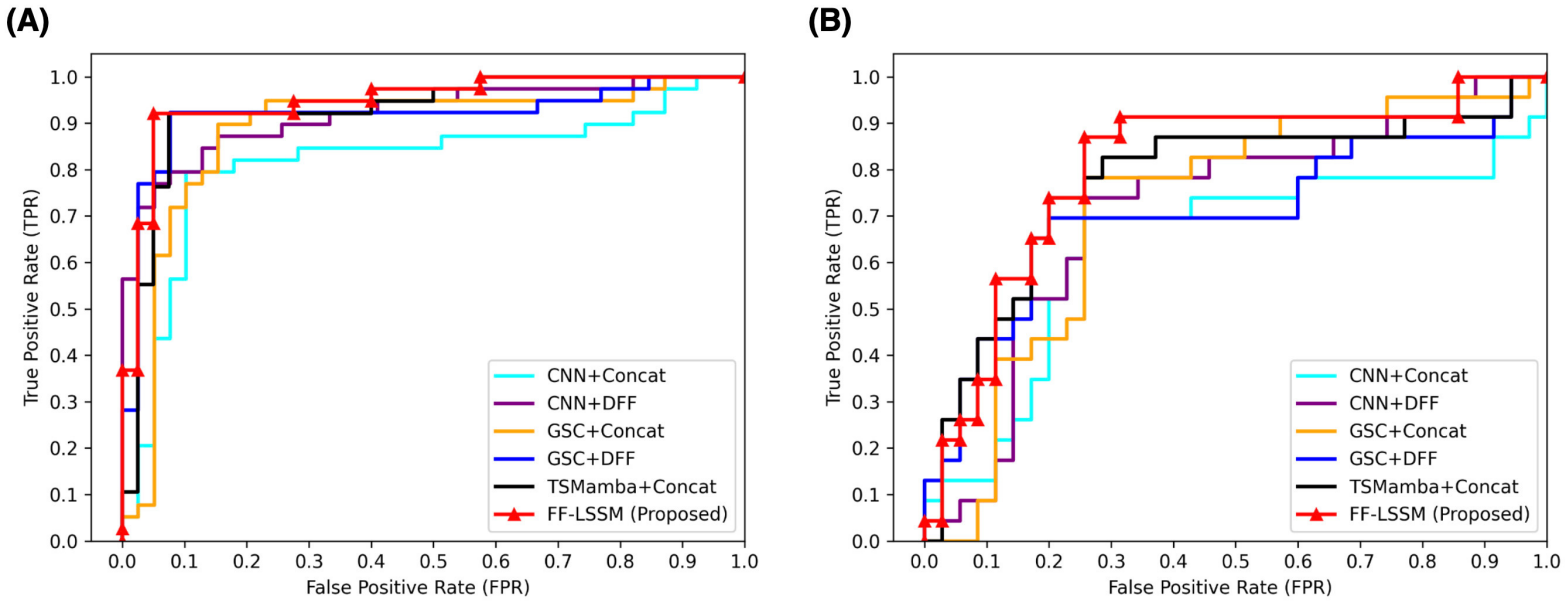
ROC curves of different methods applied in the ablation study. All models are evaluated on both **(A)** CN vs. AD and **(B)** sMCI vs. pMCI.

After verifying the efficacy of the suggested model, to validate the efficacy of the elements introduced in our model, we conducted ablation experiments. First, the effectiveness of ToM modules is tested by removing the ToM and only retaining GSC modules, resulting in the variant model GSC+DFF. Subsequently, the efficacy of the GSC module is tested by removing GSC modules and only retaining the down-sampling convolution layers, resulting in the variant model CNN+DFF. Furthermore, the efficacy of the DFF module is tested by replacing all DFF modules with concatenation operation in all models, which results in three more variants: TSMamba+Concat, GSC+Concat, and CNN+Concat. The experimental outcomes are demonstrated in Table 3, and corresponding ROC curves are illustrated in Figure 4. It is evident that the ToM module makes a substantial contribution to the model's overall efficiency when the DFF module is kept untouched. Since the ToM is the main feature extractor in the FF-LSSM and is responsible for extracting features at each level and modeling long-range dependencies, removing the ToM module leads to drastic decreases in all measures. Unsurprisingly, removing the GSC module further deteriorates the model performance since the GSC module also contributes to the feature extraction. While keeping the number of channels unchanged, the model with only down-sampling convolution layers performs the worst in both CN vs. AD and sMCI vs. pMCI tasks. This is because it is challenging to model relationships between multi-level features for a plain convolution layer. Furthermore, variant models with the simple concatenation operation always perform worse than models containing the DFF module. It can be observed that introducing the feature fusion mechanism can enhance the capability of all variant models on both classification tasks.

**Table 3:** Results of the ablation study in CN vs. AD and sMCI vs. pMCI classification tasks.

**Method**	**CN vs. AD (%)**				**sMCI vs. pMCI (%)**			
	**ACC**	**SEN**	**SPE**	**AUC**	**ACC**	**SEN**	**SPE**	**AUC**
CNN+Concat	84.62	79.49	89.74	90.86	70.69	60.87	77.14	63.73
CNN+DFF	85.90	84.62	87.18	91.49	72.41	69.57	74.29	71.30
GSC+Concat	87.18	89.74	84.62	93.16	74.14	73.91	74.29	70.56
GSC+DFF	89.74	87.18	92.31	93.03	75.86	65.22	**82.86**	72.17
TSMamba+Concat	92.31	**92.11**	92.50	**96.55**	77.59	73.91	80.00	77.39
FF-LSSM	**93.59**	**92.11**	**95.00**	96.18	**79.31**	**86.96**	74.29	**80.62**

Bold values indicate the highest value of each metric.

### 4.4 Feature visualization

**Figure 5:**
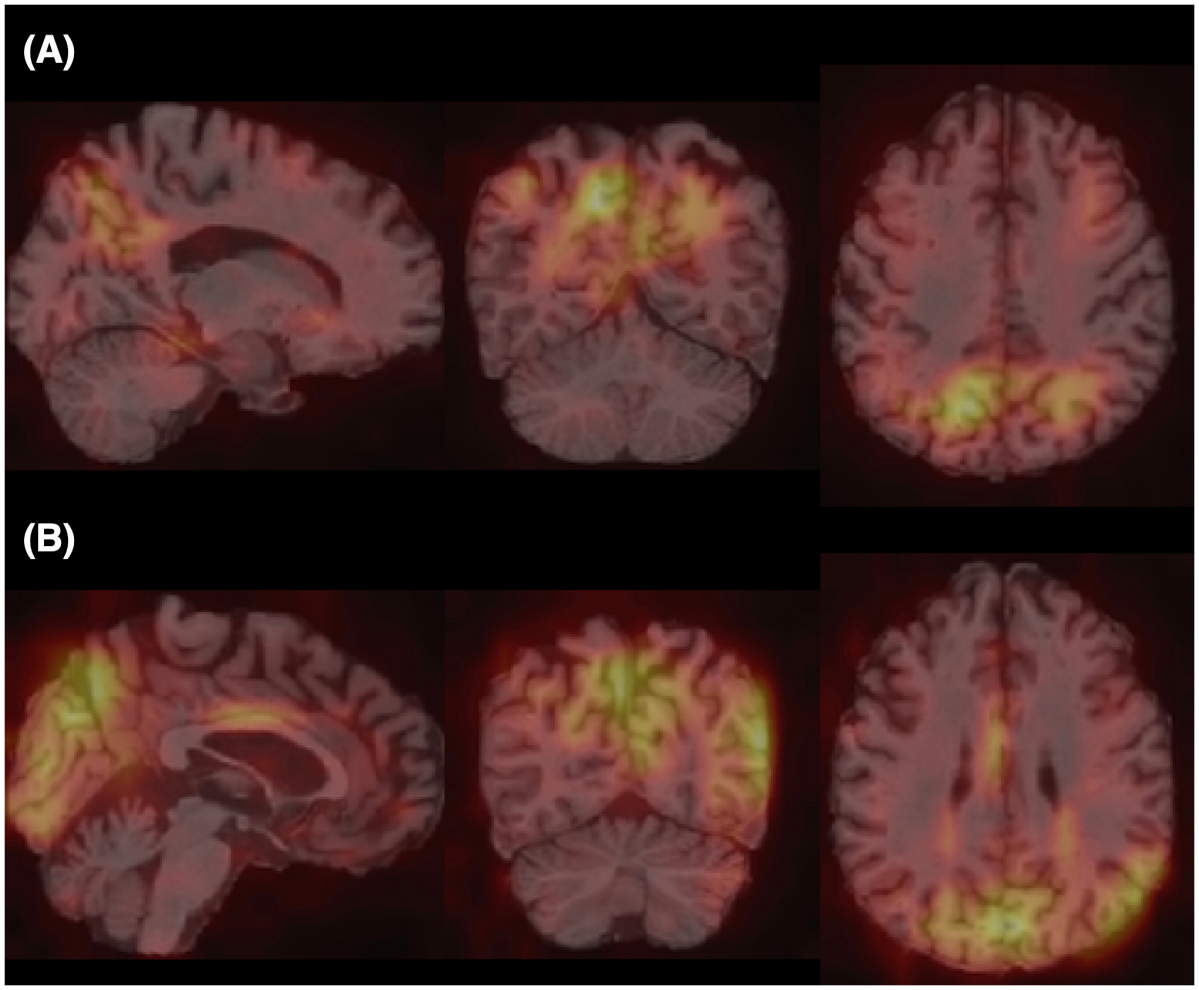
Grad-CAM heat maps obtained by **(A)** averaging over all subjects in CN vs. AD classification and **(B)** averaging over all subjects in sMCI vs. pMCI task. A brighter color represents larger gradients at the location.

**Figure 6:**
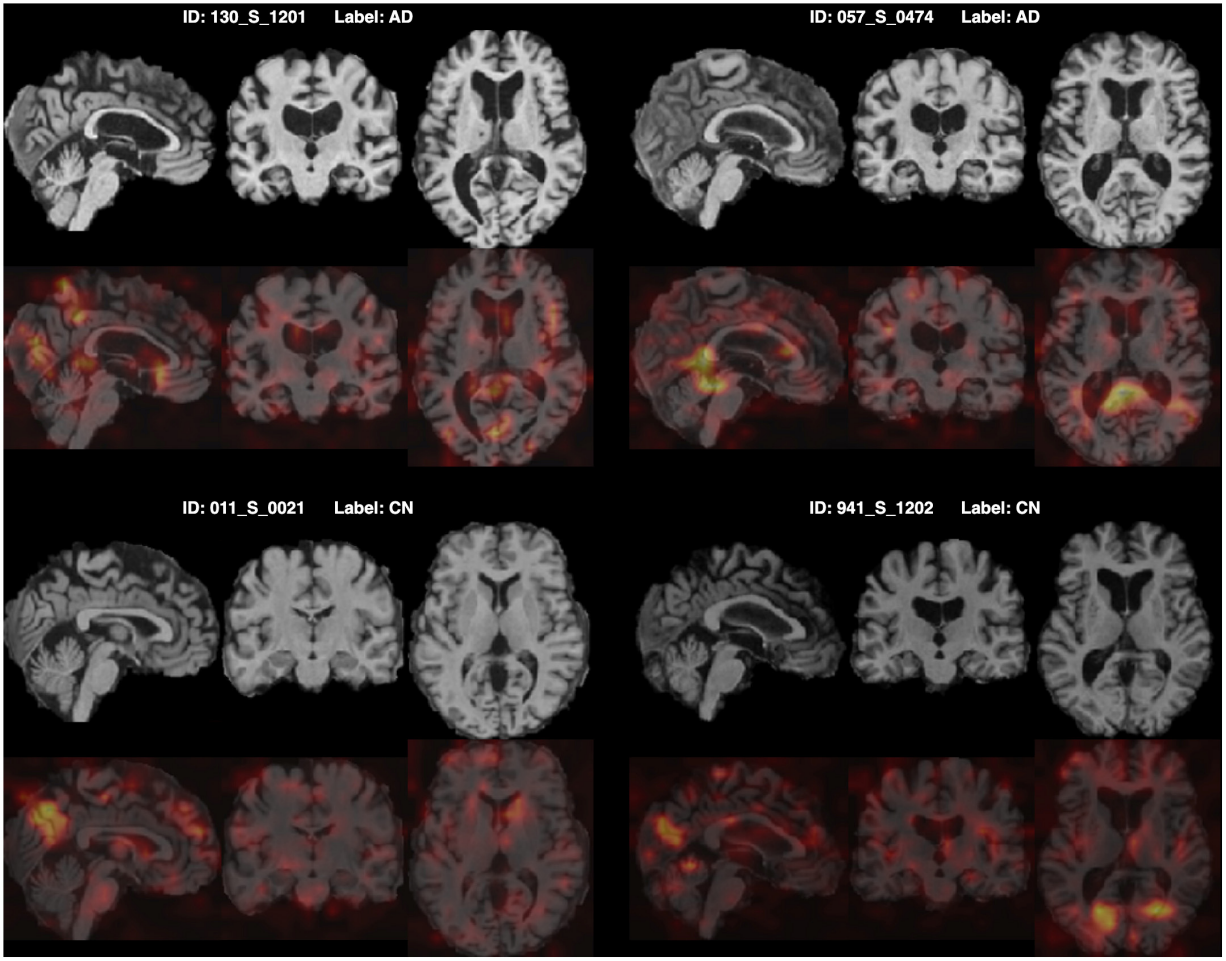
Origin MRI images and corresponding Grad-CAM heat maps of four subjects from the CN vs. AD classification.

**Figure 7:**
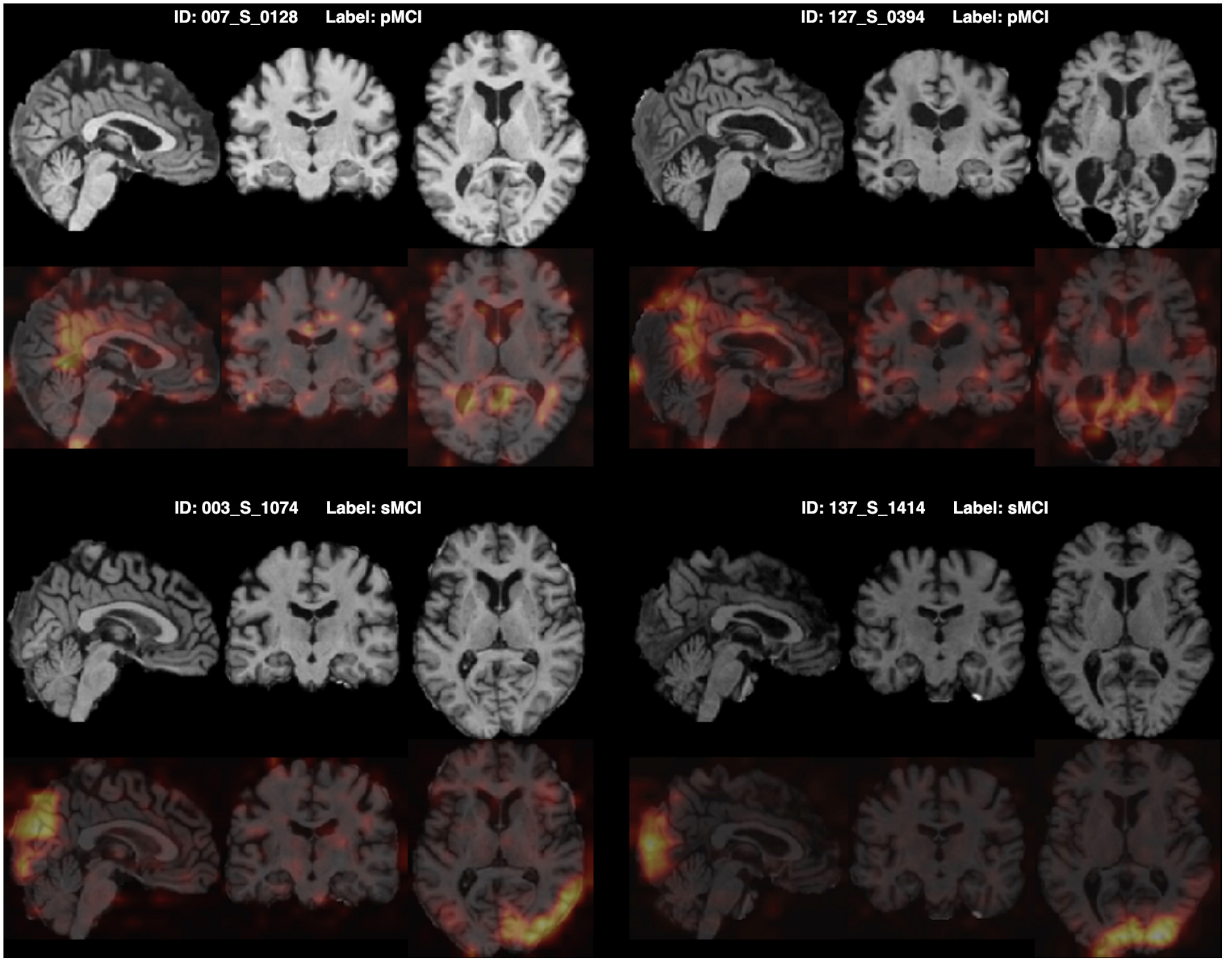
Origin MRI images and corresponding Grad-CAM heat maps of four subjects from the sMCI vs. pMCI classification.

Furthermore, for model explainability, the Grad-CAM technique is applied to figure brain areas that are most significant to the disease classifications (Selvaraju et al., 2020). Grad-CAM uses gradients of the last convolution layer and the predicted results to give a heat map visualization of why a model made its decision. It is a valuable tool for model interpretation. The Grad-CAM heat map averaged on all subjects for CN vs. AD classification is displayed in Figure 5A. It can be noticed that the implied FF-LSSM highlights many regions closely related to AD, such as the Precuneus (Karas et al., 2007), Cuneus (Zheng et al., 2025), Hippocampus (Rao et al., 2022), Caudate (Persson et al., 2018), and Lingual cortex (Liu et al., 2017). Figure 5B shows the averaged Grad-CAM for the sMCI vs. pMCI task. It comes out that the middle Cingulate, Cuneus, Precuneus, and Occipital cortex show the most significant gradients in sMCI and pMCI subjects classification, consistent with previous studies that these regions show significant volume decrease and functional abnormality in MCI progressing (Choo et al., 2010; Pagani et al., 2017; Risacher et al., 2009). In Figures 6, 7, as a reference, we also provide several individuals' MRI images and Grad-CAM heat maps from the CN vs. AD and sMCI vs. pMCI classification, respectively. This result shows that in addition to classifying dementia stages, the proposed FF-LSSM also has great potential in detecting abnormal brain regions associated with AD and MCI. This has positive significance for its future practical applications because it can provide explainable auxiliary information for diagnoses.

## 5 Discussion

With MRI and PET brain images, we build an FF-LSSM framework to extract and fuse multi-modal features for disease classifications. In Table 4, we compare our results with those reported in previous literature that also applied multi-modal methods based on MRI and PET volumes from the ADNI. The compared methods include the hierarchical feature fusion classification algorithm (Liu et al., 2014), the multiple instance-graph method (Tong et al., 2014), the multi-scale DNN (Lu et al., 2018), the multi-modal DNN with random drop-out (Forouzannezhad et al., 2018), the latent feature representation learning method (Zhou et al., 2019), the dual-modality CNN (Huang et al., 2019; Lin et al., 2021), the PT-DCN (Gao et al., 2021), and the multi-modal cross-attention AD diagnosis framework (Zhang et al., 2023). Considering most of the above studies do not provide pre-trained models or attach source codes, for comparison, we straightly use their published results in the literature. According to the results, the model we suggested outperforms previous approaches in diagnosing AD and predicting the prognosis of MCI. This is because methods used in previous studies are primarily based on ML or convolution-based layers, which cannot handle the long-range dependencies between local features. The introduction of the TSMamba module successfully solves this problem and boosts classification accuracy.

**Table 4:** Comparison with classification performance of previous multi-modal methods.

**Method**	**Subjects**				**CN vs. AD (%)**				**sMCI vs. pMCI (%)**			
	**CN**	**sMCI**	**pMCI**	**AD**	**ACC**	**SEN**	**SPE**	**AUC**	**ACC**	**SEN**	**SPE**	**AUC**
Liu et al. (2014)	229	-	-	198	82.2	77.4	86.1	88.1	-	-	-	-
Tong et al. (2014)	231	238	167	198	90.0	84.9	92.6	-	70.4	67.0	73.0	-
Lu et al. (2018)	360	409	217	238	82.9	79.7	83.8	-	-	-	-	-
Forouzannezhad et al. (2018)	248	296	193	159	89.1	87.4	92.1	-	68.2	78.1	57.5	-
Zhou et al. (2019)	204	205	157	171	-	-	-	-	74.3	-	-	75.5
Huang et al. (2019)	731	441	326	647	90.1	90.9	89.2	90.8	76.9	68.2	**83.9**	79.6
Lin et al. (2021)	308	233	183	362	92.3	90.4	94.4	92.8	74.1	75.0	73.1	76.6
Gao et al. (2021)	427	342	234	352	92.0	89.1	94.0	95.6	75.3	77.3	74.1	78.6
Zhang et al. (2023)	129	-	-	110	91.1	91.0	91.1	94.1	-	-	-	-
Ours	197	162	104	193	**93.6**	**92.1**	**95.0**	**96.2**	**79.3**	**87.0**	74.3	**80.6**

Bold values indicate the highest value of each metric.

However, the proposed model also has many limitations and there is room for further improvement in many aspects. First, only MP-RAGE MRI and 18F-FDG PET are applied in the current study. While the amalgamation of these two modalities can grant rich intelligence for dementia classification, the incorporation of other modalities, such as DTI images and genetic data, can enhance model performance to a greater degree. In addition, all experiments are performed solely on the ADNI dataset. Since different datasets usually have distinct imaging devices and acquisition parameters, migrating between them may encounter difficulties due to data diversities. Fortunately, domain adaptation and transfer learning technologies have largely solved the challenge of data heterogeneity (Zhou et al., 2023). In future studies, using multiple datasets for model training and evaluation can be more conducive to its clinical application since this scenario simulates the data heterogeneity of different medical centers. Third, due to the fine resolution of the MRI and PET images, the whole-volume feature extraction is very time-consuming. In the adopted ToM module, modeling a 64 × 64 × 64 input map can lead to a sequential length of about 260k, which is a large demand for both the time and GPU memory. Due to the limitation of GPU capacity, we only introduce two TSMamba layers in each Mamba encoder. Reducing the input resolution can reduce time and computational requirements, allowing us to stack more TSMamba layers in the encoder, but the reduced image resolution may also destroy small-scale features and worsen the model performance. Thus, the trade-off between input resolution and model scale is a direction that needs further experiments. Finally, acquiring PET modality is often more difficult than MRI due to its higher cost and potential radiation hazards. Even in large public datasets, such as ADNI, only a small fraction of the subjects have recorded PET scannings. Therefore, for multi-modality models, data sparsity is also a problem that needs to be overcome in the future. We exclusively study individuals who have both MRI and PET scans in the current study; however, to make the model more widely applicable, it is necessary to consider subjects with incomplete images. Fortunately, with the recent remarkable development of generative models (Wang S.-Q. et al., 2023; Wang et al., 2025), it has become possible to produce reliable PET images from other modalities using generative AIs. In future studies, using generative models to produce data for subjects with missing modalities will undoubtedly enhance the universality of the model.

## 6 Conclusion

For effective AD recognition, we suggest an innovative DL framework termed FF-LSSM in this study. The FF-LSSM contains two 3D Mamba encoders, one feature fusion block, and one classifier. The FF-LSSM uses MRI and PET volumes of every individual as input to conduct dementia classification by extracting and fusing multi-modal features at different scales. The experimental results show that, compared to the uni-modality model, FF-LSSM accomplishes higher classification accuracy in two binary classification tasks, which is 93.59% for the AD detection and 79.31% for the MCI prognosis, respectively. This result proves the advantage of multi-modality feature fusion and the feasibility of the suggested approach in AD detection and MCI prognosis. When juxtaposed with methods proposed by previous literature, the FF-LSSM suggested by us also achieves better classification performance. Finally, the extracted features are visualized using the Grad-CAM technique and show patterns consistent with previous studies, demonstrating the model's explainability in dementia recognition.

## Data Availability

The original contributions presented in the study are included in the article/supplementary material, further inquiries can be directed to the corresponding authors.
